# Complete Genome Sequence of an Environmental Bacillus cereus Isolate Belonging to the Bacillus anthracis Clade

**DOI:** 10.1128/MRA.00917-20

**Published:** 2020-11-19

**Authors:** Leonid M. Irenge, Bertrand Bearzatto, Jérôme Ambroise, Jean-Luc Gala

**Affiliations:** a Center for Applied Molecular Technologies, Institute of Clinical and Experimental Research, Université Catholique de Louvain, Brussels, Belgium; b Defence Laboratories Department, ACOS Ops & Trg, Belgian Armed Forces, Peutie, Belgium; University of Maryland School of Medicine

## Abstract

We report here the complete genome sequence of a Bacillus cereus isolate identified in a soil sample from Namibia. This isolate is closely related to the B. anthracis clade. While the plasmids (500 and 12 kb) carry no detectable B. anthracis virulence gene, the large plasmid shares a 50-kb continuous region similar to plasmid pXO1.

## ANNOUNCEMENT

Bacillus anthracis is the etiological cause of anthrax, a lethal disease of animals and humans (1) that can also be used as a bioweapon (2). In recent years, several B. cereus strains causing an anthrax-like disease that are phylogenetically close to B. anthracis and harbor large plasmids with sequences similar to those of B. anthracis plasmids have been characterized (3–5). Despite uncertainty about the environmental source of these clinical cases, B. cereus is a known ubiquitous soil bacterium. This study reports the entire chromosome and plasmid sequence of B. cereus CTMA-1571, isolated from a soil sample from northern Namibia, a region with a history of recurrent anthrax outbreaks in animals (6). For the isolation, 10 g of soil was ground for 1 min and homogenized in 50 ml of phosphate-buffered saline solution. After incubation under shaking for 1 h at 56°C, 0.1 ml of the solution was streaked onto a sheep blood Columbia agar plate and incubated overnight at 37°C. One of several dull-gray and opaque beta-hemolytic colonies presumably belonging to the species B. cereus was selected for further molecular characterization. This colony was grown overnight in 20 ml Luria-Bertani broth with shaking at 37°C. DNA was isolated using the phenol-chloroform protocol (7).

**FIG 1:**
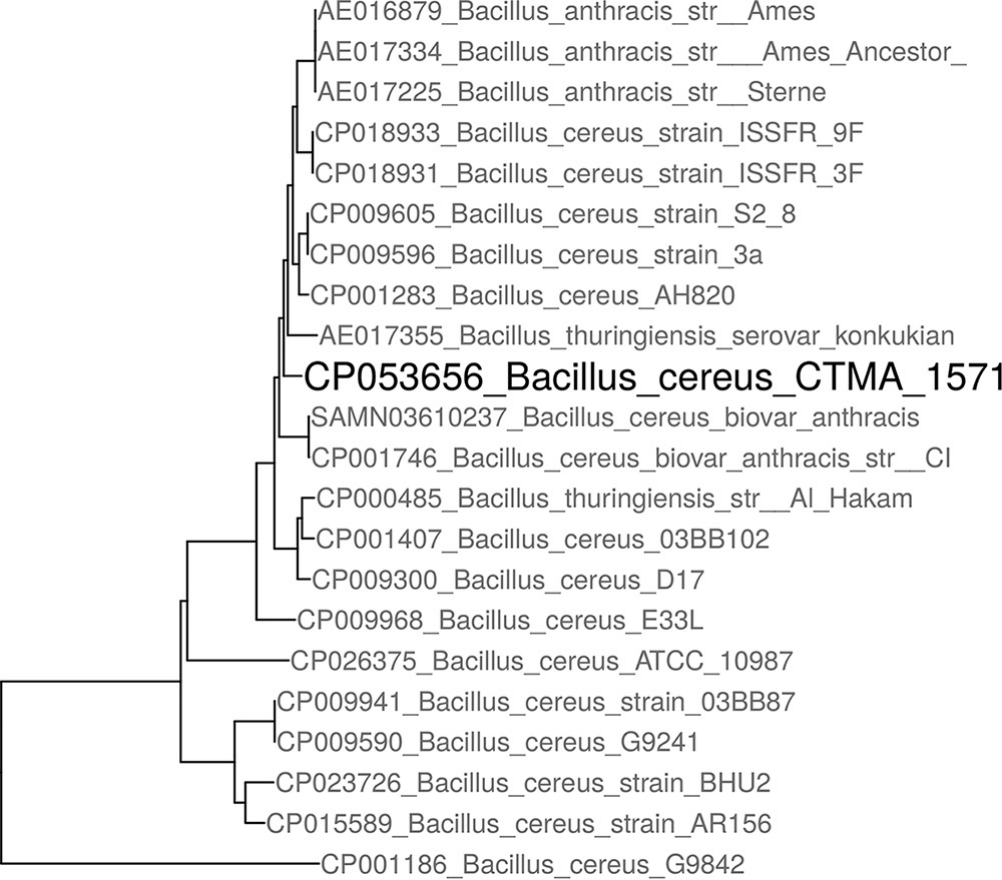
SNP-based phylogenomic tree of representative B. cereus, B. anthracis, and *B. thuringiensis* isolates.

A short-read library was prepared using a Nextera XT DNA library preparation kit (Illumina, San Diego, CA, USA) and sequenced on a MiSeq platform (Illumina) with paired-end (2 × 250-bp) reads. A total of 2 × 1,531,288 reads were generated. A long-read library was prepared using 2D genomic DNA by ligation protocol with the SQK-LSK208 2D ligation sequencing kit (Oxford Nanopore Technologies) and sequenced for 42 h on a MinION device, generating 68,046 reads with an average read length of 3,752 bp. All reads were quality checked by FastQC v.0.11.9 (http://www.bioinformatics.babraham.ac.uk/projects/fastqc/) and assembled *de novo* using SPAdes v.3.14.1 (http://cab.spbu.ru/software/spades/) (8). Terminal overlaps were aligned using AliView v.1.26 (9) and trimmed to yield circular chromosomal and plasmid sequences. The genome was rotated using the fixstart program in Circlator v.1.5.5 (10). BUSCO v.4.1.1 (11) was used to confirm the quality and completeness of the genome assembly. A single nucleotide polymorphism (SNP)-based phylogenomic tree, which was based on B. anthracis, B. thuringiensis, the closest-related B. cereus isolate, and CTMA-1571 isolates, was built using kSNP v.3.1 (12) and a k-mer size of 19. The phylogenomic tree was visualized using the ggtree R package (Fig. 1) (13). All software used default parameters unless otherwise specified. The sequences were assembled in 3 contigs. The complete genome has a total size of 5,695,148 bp, with coverages of 121× and 46× (for the Illumina and MinION data, respectively), and consists of 1 chromosome (5,182,254 bp with a G+C contents of 35.49%) and two plasmids (500,306 bp and 12,588 bp with G+C content of 32.86%, and 29.52%, respectively). Prokaryotic Genome Annotation Pipeline v.4.13 (October 2020) (14) identified a total of 5,613 coding DNA sequences, 32 rRNA sequences, and 108 tRNA sequences.

### Data availability.

The whole-genome sequence comprising the chromosome and both plasmids was deposited at DDBJ/ENA/GenBank under accession numbers CP053656 through CP053658. The raw sequence reads were deposited in the NCBI Sequence Read Archive under accession numbers SRX8842584 and SRX8842585.
